# Comparative X-ray Shielding Properties of Single-Layered and Multi-Layered Bi_2_O_3_/NR Composites: Simulation and Numerical Studies

**DOI:** 10.3390/polym14091788

**Published:** 2022-04-27

**Authors:** Arkarapol Thumwong, Jitsuna Darachai, Kiadtisak Saenboonruang

**Affiliations:** 1 Department of Materials Science, Faculty of Science, Kasetsart University, Bangkok 10900, Thailand; arkarapol.th@ku.th; 2 Department of Engineering Physics, Tsinghua University, Beijing 100084, China; jitsuna.note@gmail.com; 3 Department of Applied Radiation and Isotopes, Faculty of Science, Kasetsart University, Bangkok 10900, Thailand; 4 Kasetsart Research and Development Institute, Kasetsart University, Bangkok 10900, Thailand; 5 Specialized Center of Rubber and Polymer Materials in Agriculture and Industry (RPM), Faculty of Science, Kasetsart University, Bangkok 10900, Thailand; 6 Special Research Unit of Radiation Technology for Advanced Materials, Faculty of Science, Kasetsart University, Bangkok 10900, Thailand

**Keywords:** natural rubber, Bi_2_O_3_, X-ray shielding, simulation, multi-layered structure

## Abstract

This work theoretically compared the X-ray attenuation capabilities in natural rubber (NR) composites containing bismuth oxide (Bi_2_O_3_) by determining the effects of multi-layered structures on the shielding properties of the composites using two different software packages (XCOM and PHITS). The shielding properties of the single-layered and multi-layered Bi_2_O_3_/NR composites investigated consisted of the transmission factor (I/I_0_), effective linear attenuation coefficient (µ_eff_), effective mass attenuation coefficient (µ_m,eff_), and effective half-value layer (HVL_eff_). The results, with good agreement between those obtained from XCOM and PHITS (with less than 5% differences), indicated that the three-layered NR composites (sample#4), with the layer arrangement of pristine NR (layer#1)-Bi_2_O_3_/NR (layer#2)-pristine NR (layer#3), had relatively higher X-ray shielding properties than either a single-layer or the other multi-layered structures for all X-ray energies investigated (50, 100, 150, and 200 keV) due to its relatively larger effective percentage by weight of Bi_2_O_3_ in the composites. Furthermore, by varying the Bi_2_O_3_ contents in the middle layer (layer#2) of sample#4 from 10 to 90 wt.%, the results revealed that the overall X-ray shielding properties of the NR composites were further enhanced with additional filler, as evidenced by the highest values of µ_eff_ and µ_m,eff_ and the lowest values of I/I_0_ and HVL_eff_ observed in the 90 wt.% Bi_2_O_3_/NR composites. In addition, the recommended Bi_2_O_3_ contents for the actual production of three-layered Bi_2_O_3_/NR composites (the same layer structure as sample#4) were determined by finding the least Bi_2_O_3_ content that enabled the sample to attenuate incident X-rays with equal efficiency to that of a 0.5-mm lead sheet (with an effective lead equivalence of 0.5 mmPb). The results suggested that the recommended Bi_2_O_3_ contents in layer#2 were 82, 72, and 64 wt.% for the combined 6 mm, 9 mm, and 12 mm samples, respectively.

## 1. Introduction

Since the discovery of X-rays in 1895 by Wilhelm Roentgen, various applications have relied heavily on the utilization of X-ray technologies, especially X-ray imaging and X-ray irradiation in medicine, industry, material characterization, security, the arts, foods, and agriculture [1,2,3,4,5,6]. Despite their great potential and usefulness, excessive exposure to X-rays could harmfully affect the health of users and the public, with various symptoms, including nausea, skin burn, diarrhea, permanent disability, cancer, and death, depending on the exposure dose and duration as well as the sex, health condition, and age of those exposed [7,8]. Hence, to reduce and/or prevent the risks of excessive exposure to X-rays, a radiation safety principle, namely “As Low As Reasonably Achievable” or “ALARA”, must be strictly followed in all nuclear facilities to ensure the safety of all users and the public [9].

One of the three safety measures in ALARA is the utilization of sufficient and appropriate shielding equipment; for which different applications may require different types and specific properties from the materials [10]. For example, X-ray shielding materials based on polyethylene (PE), including Gd_2_O_3_/HDPE and nano-ZnO/HDPE composites, are suitable for applications that require exceptional strength and rigidity, such as those involving products for use as movable panels, walls, and construction parts in nuclear facilities [11,12]. On the other hand, shielding equipment, such as personal protective equipment (PPE) and covers for transporting casks, requiring exceptional flexibility, high strength, and a large amount of elongation from the materials, relies on natural and synthetic rubber composites. For example, Bi_2_O_3_/NR, Bi_2_O_3_/EPDM, BaSO_4_/EPDM, and W/SR composites were among recently developed X-ray shielding rubber materials that offered not only effective X-ray attenuation abilities but also sufficient mechanical strength and flexibility to the users [13,14,15,16]. Notably, these mentioned examples of X-ray shielding materials are lead-free, which is presently sought-after in materials, as they could substantially reduce the risks to users from exposure to highly toxic lead (Pb) elements and compounds that are common protective fillers used for the manufacturing of X-ray and gamma shielding materials due to their economical accessibility and excellent attenuation capability [17,18].

Generally, the addition of heavy metals, including Bi_2_O_3_, to the main matrix is a common method to enhance the X-ray attenuation abilities of the composites, mainly due to the relatively high atomic number (Z) and density (ρ) of Bi_2_O_3_ that enhance the interaction probabilities between the incident X-rays and the materials, subsequently increasing the ability to attenuate the incident X-rays of the composites [19]. Some examples showing the effects of Bi_2_O_3_ on improving the shielding capabilities of the composites have been reported by Intom et al., who showed that the mass attenuation coefficients (µ_m_) of Bi_2_O_3_/NR composites increased from 0.1324 to 0.3847 and then to 0.4779 cm^2^/g when the Bi_2_O_3_ contents in the NR composites increased from 0 to 80 and then to 150 parts per hundred parts of rubber by weight (phr), respectively (determined at an energy level of 223 keV) [20]. Similarly, the report from Toyen et al. suggested that increases in the Bi_2_O_3_ contents from 0 to 300 and then to 500 phr increased the linear attenuation coefficients (µ) of NR composites from 2.1 to 14.7 and then to 20.4 m^−1^, respectively (determined at an energy level of 662 keV) [13].

Nonetheless, despite the positive relationship between the contents of Bi_2_O_3_ and the shielding properties of the composites, increases in Bi_2_O_3_ contents may lead to undesirable reductions in the mechanical properties, such as decreased values of the tensile strength and elongation at the break of Bi_2_O_3_/NR composites from 14 to 7 MPa and from 630% to 500%, respectively, when the Bi_2_O_3_ contents increase from 100 to 500 phr [13]. This behavior was observed mainly due to particle agglomerations caused by filler–filler interactions and phase separation at higher filler contents [14,21]. To alleviate or limit such drawbacks by adding high filler contents to the composites, one possible method is to prepare the materials with multi-layered structures, which would enable the pristine NR layers to better support and transfer external forces exerted on the Bi_2_O_3_/NR layers, consequently limiting the reduction in the overall strength of the materials [22,23].

As aforementioned, due to the competing roles of Bi_2_O_3_ in the enhancement of X-ray-shielding properties and the reductions in mechanical properties, this work investigated appropriate multi-layered structures of Bi_2_O_3_/NR composites by theoretically comparing X-ray shielding parameters, consisting of the transmission factor (I/I_0_), the effective mass attenuation coefficient (µ_eff_), the effective linear attenuation coefficient (µ_m,eff_), the effective half-value layer (HVL_eff_), and the effective lead equivalence (Pb_eq,eff_), from 11 distinct multi-layered structures using XCOM and PHITS. In addition, the recommended Bi_2_O_3_ contents for the multi-layered structure that produced the highest shielding properties were also determined by finding the least Bi_2_O_3_ contents that, when being added to the NR composites, produced the required Pb_eq,eff_ value of 0.5 mmPb. The outcomes of this work would not only provide comparative X-ray shielding properties of multi-layered products but also present promising methods to preserve the mechanical properties of shielding materials containing high contents of fillers.

## 2. Determination of X-ray Shielding Properties Using XCOM and PHITS

### 2.1. Multi-Layered Structures of Bi_2_O_3_/NR Composites

**Figure 1:**
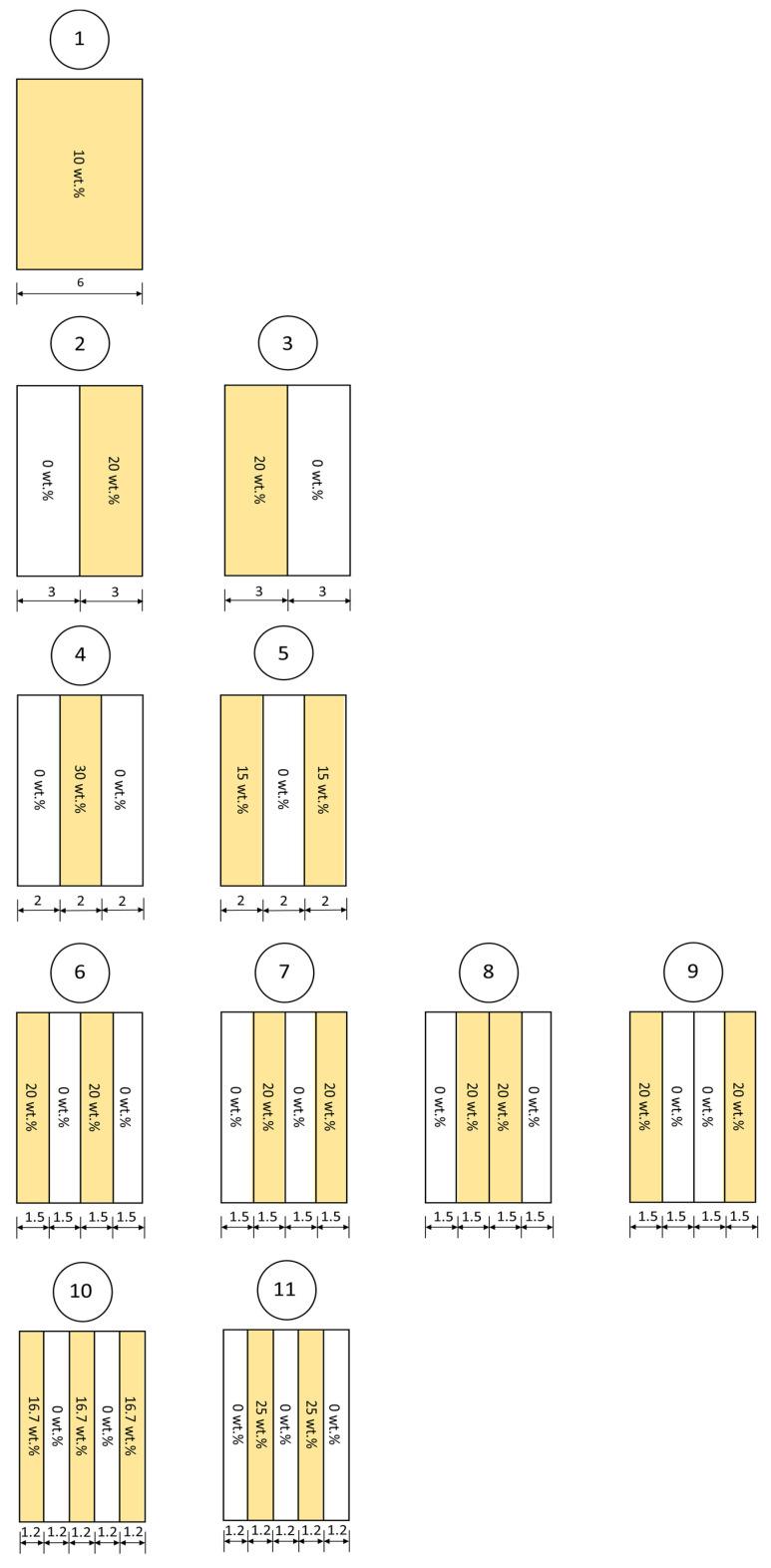
Schemes showing single-layered and multi-layered structures and Bi_2_O_3_ contents for 11 distinct designs for determination of X-ray shielding properties in Bi_2_O_3_/NR composites, where thicknesses are in millimeters and the numbers enclosed in circles represent the sample#.

The details and schemes of 11 distinct multi-layered structures for Bi_2_O_3_/NR composites with varying numbers (1–5) of layers and varying Bi_2_O_3_ contents for each layer are shown in Table 1 and Figure 1, respectively. In order to simplify the setups for the determination of X-ray shielding properties, all samples would have the same average weight contents per thickness, i.e., ΣC_i_x_i_/Σx_i_ where C_i_ and x_i_ are Bi_2_O_3_ content and thickness of the ith layer, respectively. Notably, for Figure 1, the left surface of each design was the side that faced the incident X-rays. 

### 2.2. Determination of X-ray Shielding Properties Using XCOM

The X-ray shielding properties of all 11 multi-layered structures at the X-ray energies of 50, 100, 150, and 200 keV were numerically determined using the web-based XCOM software, provided by the National Institute of Standards and Technology (NIST) (Gaithersburg, MD, USA) [24,25]. The NIST standard reference database 8 (XGAM), released in 2010, was used as the photon cross-section database in this work and the X-ray shielding parameters were calculated from the total attenuation with the inclusion of coherent scattering [26].

In order to obtain the final transmission factor (I/I_0_) for each design, the mass attenuation coefficient (µ_m_) for the Bi_2_O_3_/NR composites containing varying Bi_2_O_3_ contents of 0, 10, 15, 16.7, 20, 25, and 30 wt.% were determined using XCOM. The details of the procedure to input material parameters and contents have been described elsewhere [24]. Then, the linear attenuation coefficients (µ) for each corresponding Bi_2_O_3_ content were determined using the obtained µ_m_, following Equation (1):

(1) $$\mu =\mu_{m}\times \rho$$

where ρ is the density of the Bi_2_O_3_/NR composites containing varying Bi_2_O_3_ contents of 0, 10, 15, 16.7, 20, 25, and 30 wt.%, theoretically calculated using Equation (2):

(2) $$\rho =\frac{100}{\frac{C_{\mathrm{NR}}}{\rho_{\mathrm{NR}}}+\frac{C_{{\mathrm{Bi}}_{2}O_{3}}}{\rho_{{\mathrm{Bi}}_{2}O_{3}}}}$$

where ρ_NR_ (ρ_Bi_2_O_3__) is the density of NR (Bi_2_O_3_), which is 0.92 g/cm^3^ (8.90 g/cm^3^), and C_NR_ (C_Bi_2_O_3__) is the weight content of NR (Bi_2_O_3_) in the composites. Notably, C_NR_ + C_Bi_2_O_3__ = 100 wt.%.

The value of (I/I_0_)_i_ for the ith layer was calculated from its corresponding µ using Equation (3):

(3) $${\left(\frac{I}{I_{0}}\right)}_{i}=e^{-{\mu x}_{i}}$$

where x_i_ is the thickness of the ith layer for each design shown in Table 1. Then, the final I/I_0_ value for each sample was calculated by multiplying individual (I/I_0_)_i_ values from each layer, according to Equation (4):

(4) $$\frac{I}{I_{0}}=\prod_{1}^{n}{\left(\frac{I}{I_{0}}\right)}_{i}\;$$

where n is the number of layers in the sample and i is 1, 2, …, n.

Lastly, the effective linear attenuation coefficient (µ_eff_), the effective mass attenuation coefficient (µ_m,eff_), and the effective half-value layer (HVL_eff_), which represented the overall X-ray shielding properties for each design, were determined using Equations (5)–(7), respectively:

(5) $$\mu_{\mathrm{eff}}=\frac{-\ln{\left(\frac{I}{I_{0}}\right)}_{i}}{x_{i}}$$

(6) $${µ}_{m,\mathrm{eff}}=\frac{\mu_{\mathrm{eff}}}{\rho_{\mathrm{eff}}}$$

(7) $${\mathrm{HVL}}_{\mathrm{eff}}=\frac{\ln\left(2\right)}{\mu_{\mathrm{eff}}}$$

where ρ_eff_ is the effective density of the sample, calculated using Equation (8):

(8) $$\rho_{\mathrm{eff}}=\frac{\sum_{1}^{n}\rho_{i}x_{i}}{\sum_{1}^{n}x_{i}}$$

where ρ_i_ and x_i_ are the density and the thickness of the ith layer, respectively. Notably, for further determination, the values of µ for a pure Pb sheet at X-ray energies of 50, 100, 150, and 200 keV were also determined using XCOM. Furthermore, the effective percentage by weight (C_eff,Bi_2_O_3__) of Bi_2_O_3_ in different multi-layered samples (sample#2–sample#11) was also determined using Equation (9), which was derived from Equation (2):

(9) $$\rho_{\mathrm{eff}}=\frac{100}{\frac{100-C_{\mathrm{eff},{\mathrm{Bi}}_{2}O_{3}}}{\rho_{\mathrm{NR}}}+\frac{C_{\mathrm{eff},{\mathrm{Bi}}_{2}O_{3}}}{\rho_{{\mathrm{Bi}}_{2}O_{3}}}}$$

### 2.3. Determination of X-ray Shielding Properties Using PHITS

In order to verify the X-ray shielding properties obtained using XCOM, the final I/I_0_ values were also determined for all multi-layered structures using PHITS by setting up the incident X-ray beam with a diameter of 1 mm pointing directly to the center of each sample, having a surface area of 20 cm × 20 cm and a combined thickness of 6 mm. This setup would minimize the possible overestimation of the final I/I_0_ value caused by build-up effects [27]. In addition, the detector with a 100% detection efficiency was set up to capture all primary transmitted X-rays. Further details of the PHITS setup are provided elsewhere [10,11]. The percentages of difference (%Difference) between the final I/I_0_ values obtained from XCOM and those from PHITS were determined, following Equation (10):

(10) $$\%\mathrm{Difference}=\frac{\left|{\left(\frac{I}{I_{0}}\right)}_{\mathrm{XCOM}}-{\left(\frac{I}{I_{0}}\right)}_{\mathrm{PHITS}}\right|}{{\left(\frac{I}{I_{0}}\right)}_{\mathrm{XCOM}}}\times 100\%$$

where (I/I_0_)_XCOM_ and (I/I_0_)_PHITS_ are the effective transmission factors of the Bi_2_O_3_/NR composites obtained from XCOM and PHITS, respectively.

### 2.4. Determination of Effective Lead Equivalence and Recommended Contents of Bi_2_O_3_

The values of effective lead equivalence (Pb_eq,eff_) at X-ray energies of 50, 100, 150, and 200 keV for the multi-layered Bi_2_O_3_/NR composites offering the highest final I/I_0_ values among all 11 designs were calculated, following Equation (11):

(11) $$\mu_{\mathrm{Pb}}{\mathrm{Pb}}_{\mathrm{eq},\mathrm{eff}}={µ}_{\mathrm{NR},\mathrm{eff}}x_{\mathrm{NR}}$$

where µ_Pb_ is the linear attenuation coefficient of a pure Pb sheet, µ_NR,eff_ is the effective linear attenuation coefficient of multi-layered Bi_2_O_3_/NR composites, and x_NR_ is the combined thickness of the multi-layered Bi_2_O_3_/NR composites, which varied from 6 to 9 to 12 mm. Notably, the Bi_2_O_3_ contents for the determination of Pb_eq,eff_ were varied up to the maximum content of 90 wt.% and the µ_Pb_ values were 90.9, 62.7, 22.8, and 1.13 cm^−1^ at X-ray energies of 50, 100, 150, and 200 keV, respectively, determined using XCOM.

To determine the recommended Bi_2_O_3_ contents, the values of Pb_eq,eff_ for all conditions obtained from the previous steps were plotted against their corresponding Bi_2_O_3_ contents. Then, a horizontal straight line with a Pb_eq_ value of 0.5 mmPb (the common requirement for X-ray shielding equipment in general nuclear facilities) was plotted and the points of intersection were noted for each thickness (6, 9, and 12 mm), which represented the least Bi_2_O_3_ contents providing the composites with a Pb_eq_ value of 0.5 mmPb, and could be regarded as the recommended Bi_2_O_3_ contents for the actual production.

## 3. Results and Discussion

### 3.1. Values of µ_m_, µ, and ρ for Bi_2_O_3_/NR Composites

The values of the numerically determined µ_m_, ρ, and µ for the single-layered Bi_2_O_3_/NR composites with varying Bi_2_O_3_ contents of 0, 10, 15, 16.7, 20, 25, or 30 wt.% at X-ray energies of 50, 100, 150, and 200 keV are shown in Table 2, Table 3 and Table 4, respectively. The results shown in Table 2 indicated that the values of µ_m_ tended to increase with increasing Bi_2_O_3_ content but decreased with increasing X-ray energy. The positive relationship between µ_m_ and filler contents was mainly due to the high atomic number (Z) of Bi and the much higher density (ρ) of Bi_2_O_3_ compared to those of NR, resulting in substantially enhanced interaction probabilities between the incident X-rays and the materials through the very effective and dominant X-ray interaction, namely photoelectric absorption, which subsequently improved the overall X-ray shielding properties of the composites with the addition of Bi_2_O_3_. The behavior could be mathematically explained by considering the relationship between the photoelectric cross-section (σ_pe_), atomic numbers (Z) of elements in the composites, and the frequencies (ν) of incident X-rays, following Equation (12):

(12) $$\sigma_{\mathrm{pe}}=\frac{Z^{n}}{{\left(h\nu \right)}^{3}}$$

where h is Planck’s constant [11].

Notably, ν and the X-ray energy (E) are directly proportional to each other as shown in Equation (13):

(13) E=hν

Equations (12) and (13) also depict that the interaction probabilities between the incident X-rays and the materials are inversely proportional to ν^3^ or E^3^; for which the results in Table 2 clearly illustrate this effect, as evidenced by the lowest µ_m_ values being observed at the X-ray energy of 200 keV [28].

Table 3, which shows the calculated densities (ρ) of a single-layered Bi_2_O_3_/NR composite with varying Bi_2_O_3_ contents of 0, 10, 15, 16.7, 20, 25, and 30 wt.% that were used for the determination of the linear attenuation coefficient (µ), suggested that the density of the NR composites increased with increasing Bi_2_O_3_ contents, which is mainly due to the much higher ρ value of Bi_2_O_3_ (ρ_Bi_2_O_3__ = 8.90 g/cm^3^) than for NR (ρ_NR_ = 0.92 g/cm^3^). Using the results shown in Table 2 and Table 3, and Equation (1), the values of µ for all the single-layered Bi_2_O_3_/NR composites with varying Bi_2_O_3_ contents of 0, 10, 15, 16.7, 20, 25, and 30 wt.% were determined, and the results are shown in Table 4, which indicates similar behavior as for µ_m_ (Table 2). However, more pronounced effects of Bi_2_O_3_ on the enhancement of µ were observed compared to those for µ_m_ due to the simultaneous roles of Bi_2_O_3_ in increasing both the µ_m_ and ρ values of the composites, which further amplified the values of µ at higher Bi_2_O_3_ contents (Equation (1)).

### 3.2. Final I/I_0_ of Multi-Layered Bi_2_O_3_/NR Composites

**Figure 2:**
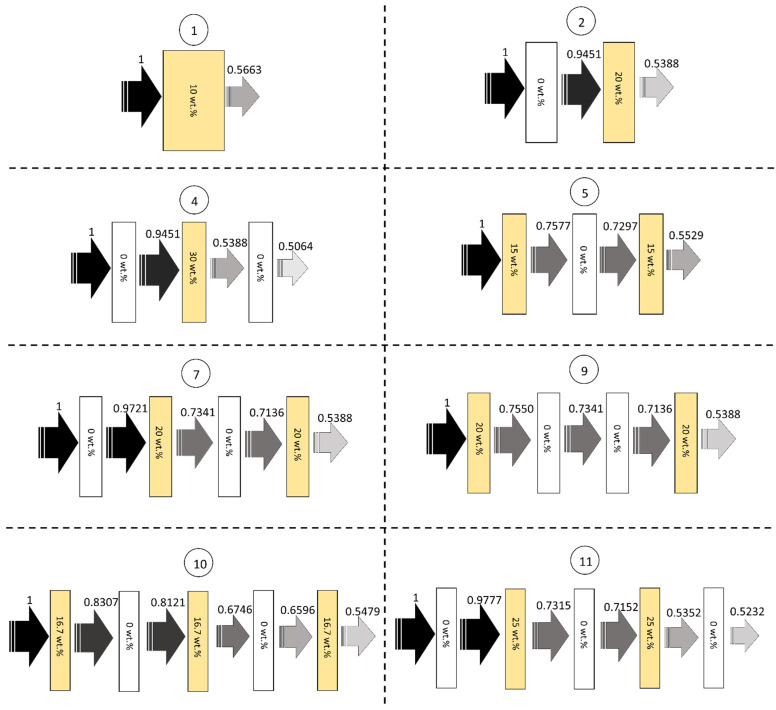
Schemes showing relative X-ray intensities for each layer of sample#1, sample#2, sample#4, sample#5, sample#7, sample#9, sample#10, and sample#11, at the X-ray energy of 50 keV. The numbers enclosed in circles represent sample#.

Table 5, Table 6, Table 7 and Table 8 show the transmission factors (I/I_0_) for each layer as well as the final I/I_0_ values of the 11 multi-layered Bi_2_O_3_/NR composites at X-ray energies of 50, 100, 150, and 200 keV, respectively, and Figure 2 shows the schematic representation of relative X-ray intensities for each layer of some designs at the X-ray energy of 50 keV. All the results suggested that the NR layers containing Bi_2_O_3_ could attenuate X-rays with higher efficiencies than those without Bi_2_O_3_ due to the much higher µ values of Bi_2_O_3_/NR composites (Table 4), especially those with higher Bi_2_O_3_ contents, that better interacted and attenuated incident X-rays. Furthermore, the results revealed that the final I/I_0_ values for the composites had larger transmitted X-ray intensities at higher X-ray energies (for the same sample#). This behavior could be explained using Equation (12), which suggested that the interaction probabilities, as well as their X-ray attenuation capabilities, decreased with increasing X-ray energies, resulting in more X-rays being able to escape the materials.

Among the 11 multi-layered designs, sample#4, which has a three-layered structure, had the lowest final I/I_0_ values of 0.5064, 0.6178, 0.7995, and 0.8645 at X-ray energies of 50, 100, 150, and 200 keV, respectively, while sample#1, a single-layered structure, had the highest final I/I_0_ values of 0.5663, 0.6671, 0.8220, and 0.8765 at X-ray energies of 50, 100, 150, and 200 keV, respectively. Based on the results from these two designs, the multi-layered structure exhibited higher X-ray shielding capabilities by as much as 10.5, 8.7, 4.0, and 2.1% compared to a single-layered structure, determined at X-ray energies of 50, 100, 150, and 200 keV, respectively. Specifically, for sample#4, its highest X-ray attenuation capability was due to its highest effective density and effective percentage by weight of Bi_2_O_3_ contained in the sample, determined using Equations (8) and (9); for which the results of both parameters for all designs are shown in Table 9. The larger values of both quantities in multi-layered structures were mainly due to the much higher density of Bi_2_O_3_ particles in comparison with that of the NR matrix (for instance, adding 20 wt.% of Bi_2_O_3_ to layer#2 in sample#4 would require much less volume than removing 20 wt.% of NR, resulting in a considerable reduction in the total volume and subsequently the increase in the density of the sample). These effects then enabled sample#4 to have more Bi atoms to interact with incoming X-rays through the photoelectric absorption than that of sample#1.

In addition, Equation (3) could be modified for the calculation of I/I_0_ as Equation (14):

(14) $$\frac{I}{I_{0}}=e^{-\overset{N}{\underset{i}{\sum }}\mu_{i}x_{i}}$$

where µ_i_ is the linear attenuation coefficient of the ith layer, x_i_ is the thickness of the ith layer, and N is the total number of layers in the composites [29], which depicted that the values of $\overset{N}{\underset{i}{\sum }}\mu_{i}x_{i}$ for the multi-layered structures (using information from Table 1 and Table 4) were larger than that of the single-layered sample. For instance, sample#4 had the value of $\overset{N}{\underset{i}{\sum }}\mu_{i}x_{i}$ of 0.6804, while sample#1 had the value of 0.5687, leading to a lower I/I_0_ and better X-ray shielding capabilities in sample#4. Furthermore, the results showed that rearranging layers of the samples having the same Bi_2_O_3_ contents and numbers of layers did not have effects on X-ray shielding capabilities. For instance, sample #2 and sample #3, as well as samples #6–#9, had the same values of I/I_0_, regardless of how the layers were arranged. This was due to the values of $\overset{N}{\underset{i}{\sum }}\mu_{i}x_{i}$ being the same for all of them.

Table 10 shows the final I/I_0_ values of all 11 multi-layered structures using XCOM and PHITS, as well as their corresponding %Difference values for these two methods. The comparisons indicated that the results obtained from both methods were in good agreement, with the largest %Difference value being 4.78% and the average %Difference being 2.24%. Consequently, the values obtained from XCOM and PHITS could be further used for the determination of other parameters, including µ_m,eff_, µ_eff_, HVL_eff_, and Pb_eq,eff_. Another interesting outcome from Table 9 was that the final I/I_0_ values from PHITS seemed to be slightly higher than those from XCOM. This could have been due to factors, such as backscattering and the rescattering of X-rays inside the materials, resulting in an increase in the transmitted X-rays and a subsequent underestimation of the theoretical or ideal X-ray attenuation capabilities of the composites in the results obtained from PHITS [30].

### 3.3. Values for µ_eff_, µ_m,eff_, and HVL_eff_ of Multi-Layered Bi_2_O_3_/NR Composites

Table 11 shows the values of µ_eff_, µ_m,eff_, and HVL_eff_ for the 11 multi-layered Bi_2_O_3_/NR composites at X-ray energies of 50, 100, 150, and 200 keV, determined using Equations (5)–(7) and the effective densities (ρ_eff_) of the samples shown in Table 9. The results indicated that similar to those of the final I/I_0_ (Table 10), sample#4 had the most efficient X-ray shielding properties as well as ρ_eff_, as evidenced by its highest values of µ_eff_, µ_m,eff_, and HVL_eff_ compared to the other designs.

### 3.4. X-rays Shielding Properties and Recommended Bi_2_O_3_ Contents of Three-Layered Bi_2_O_3_/NR Composites (Sample#4)

**Figure 3:**
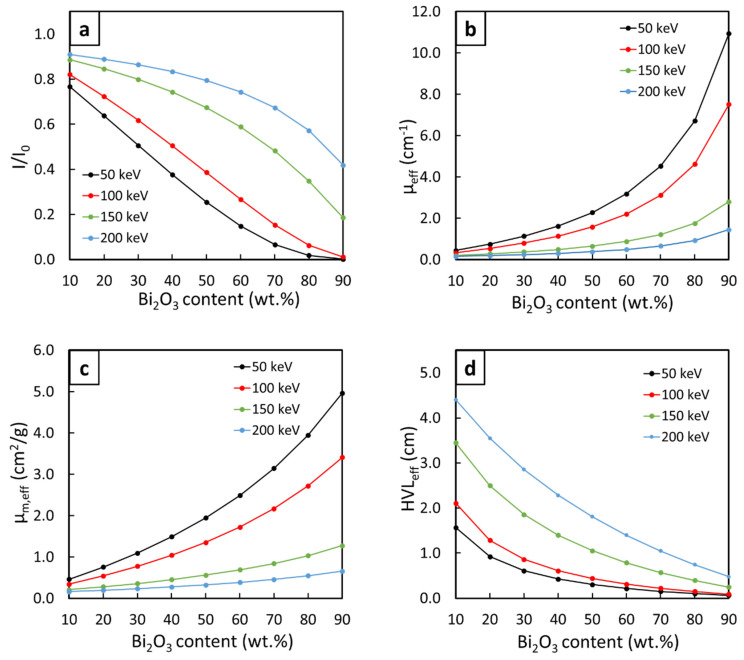
(**a**) Final values of I/I_0_, (**b**) µ_eff_, (**c**) µ_m,eff_, and (**d**) HVL_eff_ of three-layered Bi_2_O_3_/NR composites (sample#4) containing varying Bi_2_O_3_ contents from 10 to 90 wt.% in layer#2 (middle layer) and a fixed combined thickness of 6 mm, determined at X-ray energies of 50, 100, 150, and 200 keV.

Figure 3 shows the values of the final I/I_0_, µ_eff_, µ_m,eff_, and HVL_eff_ of the three-layered Bi_2_O_3_/NR composites (sample#4, which provided higher X-ray shielding properties compared to the other designs), with varying Bi_2_O_3_ contents in layer#2 (middle layer) from 10 to 90 wt.% in 10 wt.% increments and a fixed combined thickness of 6 mm, determined at X-ray energies of 50, 100, 150, and 200 keV. The results indicated that the ability to attenuate incident X-rays greatly improved with increasing Bi_2_O_3_ contents, as evidenced by the decreases in the values of I/I_0_ and HVL_eff_ and the increases in µ_eff_ and µ_m,eff_ with increasing contents. On the other hand, the overall shielding properties of the composites tended to decrease with increasing X-ray energy, as the lowest (highest) values of µ_eff_ and µ_m,eff_ (I/I_0_ and HVL_eff_) were observed at an X-ray energy of 200 keV. These two sets of behavior could be explained using Equation (12), which states that the photoelectric cross-section (σ_pe_) (the ability to attenuate X-rays) is directly proportional to Z^n^ while being inversely proportional to ν^3^ (E^3^), resulting in enhanced (lower) shielding properties at higher filler contents (X-ray energies).

**Figure 4:**
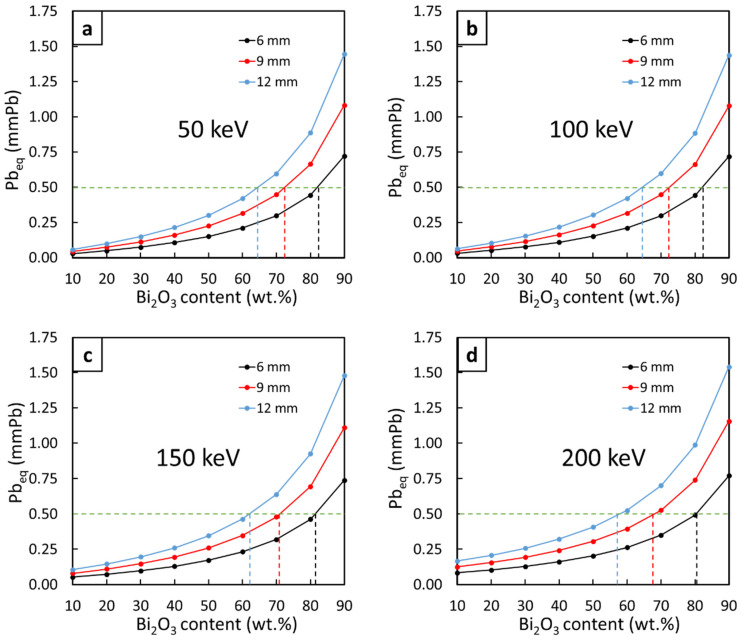
Effective Pb_eq_ of three-layered Bi_2_O_3_/NR composites (sample#4) with varying Bi_2_O_3_ contents from 10 to 90 wt.% in layer#2 (middle layer) and varying combined thicknesses of 6, 9, and 12 mm, determined at X-ray energies of (**a**) 50, (**b**) 100, (**c**) 150, and (**d**) 200 keV. The green dotted lines represent the common requirement of 0.5 mmPb used as a benchmark for this work and the blue, red, and black dotted lines represent the least Bi_2_O_3_ contents providing the composites with the Pb_eq_ of the required 0.5 mmPb for varying thicknesses.

The Pb_eq,eff_ values of the three-layered Bi_2_O_3_/NR composites (sample#4) with varying Bi_2_O_3_ contents in layer#2 (middle layer) from 10 to 90 wt.% in 10 wt.% increments and varying combined thicknesses of 6, 9, and 12 mm, are shown in Figure 4. The results indicated that the least Bi_2_O_3_ contents in layer#2, which could be regarded as the recommended Bi_2_O_3_ contents, that provided the three-layered NR composites with the required Pb_eq_ of 0.5 mmPb, were 82, 72, and 64 wt.% for the combined thicknesses of 6, 9, and 12 mm, respectively. The decreases in the recommended Bi_2_O_3_ contents with thicker samples were due to more Bi atoms being available in thicker materials (with the same filler content) to interact with incident X-rays, subsequently reducing the required Bi_2_O_3_ contents in layer#2. Notably, while it is possible to prepare NR composites with a 90 wt.% of fillers, as reported by Gwaily et al. who prepared Pb/NR composites for gamma shielding with the Pb contents up to 2000 phr (~95 wt.%) [31], difficulties in the sample preparation process, as well as possible substantial reductions in mechanical properties, could limit the processibility of multi-layered composites with very high filler contents. Consequently, for applications that allow space for thicker materials, lower recommended Bi_2_O_3_ fillers, such as those in 9 mm and 12 mm samples, should be considered to ease the difficulty and preserve the mechanical properties and product flexibility.

In order to understand how the developed multi-layered structure (sample#4) performed with respect to previously reported materials, the results revealed that sample#4 in this work with the Bi_2_O_3_ content of 90 wt.% in layer#2 (middle layer) exhibited the µ value of 7.51 cm^−1^ (at 100 keV), while the dimensionally-enhanced wood/Bi_2_O_3_/NR composites and Gd_2_O_3_/NR composites with a total Bi_2_O_3_ content of 50 phr (approximately equal Bi_2_O_3_ content in the sample as those in sample#4) but with a single-layer structure, had the µ values of 2–3 and 2.6 cm^−1^ (at 100 keV), respectively [11,32]. These comparisons clearly indicate that the use of a multi-layered structure had great potential to substantially improve the X-ray shielding properties of the products.

## 4. Conclusions

This work theoretically compared the X-ray shielding properties of single-layered and multi-layered Bi_2_O_3_/NR composites by determining various shielding parameters (µ_eff_, µ_m,eff_, HVL_eff_, and Pb_eq,eff_). In total, 11 different single-layered and multi-layered designs were used to investigate the X-ray attenuation capabilities at X-ray energies of 50, 100, 150, and 200 keV. The results indicated that the layers with higher Bi_2_O_3_ contents had better shielding abilities than those with lower contents and the three-layered structure (sample#4), with the layer arrangement of pristine NR (layer#1)-Bi_2_O_3_/NR (layer#2)-pristine NR (layer#3), had the highest overall X-ray shielding properties among the designs investigated, due to its highest effective Bi_2_O_3_ content, offering enhanced X-ray shielding properties of 10.5, 8.7, 4.0, and 2.1% compared to those of a single-layered structure (sample#1). Additionally, further investigation by varying the Bi_2_O_3_ contents in layer#2 (the middle layer) of sample#4 from 10 to 90 wt.% in 10 wt.% increments revealed that the X-ray shielding properties could be further enhanced by increasing the Bi_2_O_3_ contents; for which the recommended filler contents for actual production, determined from the common required Pb_eq_ value of 0.5 mm Pb, were 82, 72, and 64 wt.% for sample combined thicknesses of 6, 9, and 12 mm, respectively. The overall outcomes of this work reported not only the comparison of X-ray shielding properties of single-layered and multi-layered Bi_2_O_3_/NR composites but also presented potential methods to limit the reduction in mechanical properties and flexibility of the composites containing high filler contents.

## Figures and Tables

**Table 1:** Sample codes with details of the number of layers, thickness of each layer, and Bi_2_O_3_ content in each layer for determination of X-ray shielding properties in Bi_2_O_3_/NR composites (Sample# and Layer# denote Sample Number and Layer Number, respectively).

Sample#	Number of Layers	Thickness of Each Layer (mm)	Bi_2_O_3_ Contents in Layer# (wt.%)				
			1	2	3	4	5
1	1	6.0	10	-	-	-	-
2	2	3.0	0	20	-	-	-
3	2	3.0	20	0	-	-	-
4	3	2.0	0	30	0	-	-
5	3	2.0	15	0	15	-	-
6	4	1.5	20	0	20	0	-
7	4	1.5	0	20	0	20	-
8	4	1.5	0	20	20	0	-
9	4	1.5	20	0	0	20	-
10	5	1.2	16.7	0	16.7	0	16.7
11	5	1.2	0	25	0	25	0

**Table 2:** Mass attenuation coefficients (µ_m_; cm^2^/g) of Bi_2_O_3_/NR composites with varying Bi_2_O_3_ contents of 0, 10, 15, 16.7, 20, 25, and 30 wt.% at the X-ray energies of 50, 100, 150, and 200 keV.

X-ray Energy (keV)	Bi_2_O_3_ Content (wt.%)						
	0	10	15	16.7	20	25	30
50	0.2047	0.9379	1.3050	1.4290	1.6710	2.0380	2.4040
100	0.1683	0.6677	0.9174	1.0020	1.1670	1.4170	1.6670
150	0.1501	0.3233	0.4099	0.4393	0.4965	0.5831	0.6696
200	0.1371	0.2174	0.2575	0.2712	0.2976	0.3378	0.3779

**Table 3:** Calculated densities (ρ) of Bi_2_O_3_/NR composites with varying Bi_2_O_3_ contents of 0, 10, 15, 16.7, 20, 25, and 30 wt.%.

Bi_2_O_3_ Content (wt.%)	Density (g/cm^3^)
0	0.920
10	1.011
15	1.063
16.7	1.082
20	1.121
25	1.186
30	1.259

**Table 4:** Linear attenuation coefficients (µ; cm^−1^) of single-layered Bi_2_O_3_/NR composites with varying Bi_2_O_3_ contents of 0, 10, 15, 16.7, 20, 25, and 30 wt.% at X-ray energies of 50, 100, 150, and 200 keV.

X-ray Energy (keV)	Bi_2_O_3_ Content (wt.%)						
	0	10	15	16.7	20	25	30
50	0.1883	0.9478	1.3872	1.5457	1.8732	2.4167	3.0255
100	0.1548	0.6747	0.9751	1.0838	1.3082	1.6803	2.0979
150	0.1380	0.3267	0.4357	0.4751	0.5565	0.6914	0.8427
200	0.1261	0.2197	0.2737	0.2933	0.3336	0.4005	0.4755

**Table 5:** Relative X-ray intensities for each layer of multi-layered Bi_2_O_3_/NR composites at an X-ray energy of 50 keV (Sample# and Layer# denote Sample Number and Layer Number, respectively).

Sample#	Number of Layers	Thickness of Each Layer (mm)	Relative X-ray Intensities for Layer#				
			1	2	3	4	5
1	1	6.0	0.5663	-	-	-	-
2	2	3.0	0.9451	0.5388	-	-	-
3	2	3.0	0.5701	0.5388	-	-	-
4	3	2.0	0.9630	0.5258	0.5064	-	-
5	3	2.0	0.7577	0.7298	0.5529	-	-
6	4	1.5	0.7550	0.7340	0.5542	0.5388	-
7	4	1.5	0.9722	0.7340	0.7136	0.5388	-
8	4	1.5	0.9722	0.7340	0.5542	0.5388	-
9	4	1.5	0.7550	0.7340	0.7136	0.5388	-
10	5	1.2	0.8307	0.8121	0.6746	0.6596	0.5479
11	5	1.2	0.9777	0.7315	0.7152	0.5352	0.5232

**Table 6:** Relative X-ray intensities for each layer of multi-layered Bi_2_O_3_/NR composites at an X-ray energy of 100 keV (Sample# and Layer# denote Sample Number and Layer Number, respectively).

Sample#	Number of Layers	Thickness of Each Layer (mm)	Relative X-ray Intensities for Layer#				
			1	2	3	4	5
1	1	6.0	0.6671	-	-	-	-
2	2	3.0	0.9546	0.6447	-	-	-
3	2	3.0	0.6754	0.6447	-	-	-
4	3	2.0	0.9695	0.6373	0.6178	-	-
5	3	2.0	0.8228	0.7977	0.6564	-	-
6	4	1.5	0.8218	0.8030	0.6599	0.6447	-
7	4	1.5	0.9770	0.8030	0.7845	0.6447	-
8	4	1.5	0.9770	0.8030	0.6599	0.6447	-
9	4	1.5	0.8218	0.8030	0.7845	0.6447	-
10	5	1.2	0.8780	0.8619	0.7568	0.7428	0.6522
11	5	1.2	0.9816	0.8023	0.7876	0.6438	0.6319

**Table 7:** Relative X-ray intensities for each layer of multi-layered Bi_2_O_3_/NR composites at an X-ray energy of 150 keV (Sample# and Layer# denote Sample Number and Layer Number, respectively).

Sample#	Number of Layers	Thickness of Each Layer (mm)	Relative X-ray Intensities for Layer#				
			1	2	3	4	5
1	1	6.0	0.8220	-	-	-	-
2	2	3.0	0.9594	0.8119	-	-	-
3	2	3.0	0.8462	0.8119	-	-	-
4	3	2.0	0.9728	0.8219	0.7995	-	-
5	3	2.0	0.9165	0.8916	0.8172	-	-
6	4	1.5	0.9199	0.9010	0.8289	0.8119	-
7	4	1.5	0.9795	0.9010	0.8826	0.8119	-
8	4	1.5	0.9795	0.9010	0.8289	0.8119	-
9	4	1.5	0.9199	0.9010	0.8826	0.8119	-
10	5	1.2	0.9446	0.9291	0.8776	0.8631	0.8153
11	5	1.2	0.9836	0.9052	0.8904	0.8195	0.8060

**Table 8:** Relative X-ray intensities for each layer of multi-layered Bi_2_O_3_/NR composites at an X-ray energy of 200 keV (Sample# and Layer# denote Sample Number and Layer Number, respectively).

Sample#	Number of Layers	Thickness of Each Layer (mm)	Relative X-ray intensities for Layer#				
			1	2	3	4	5
1	1	6.0	0.8765	-	-	-	-
2	2	3.0	0.9629	0.8712	-	-	-
3	2	3.0	0.9048	0.8712	-	-	-
4	3	2.0	0.9751	0.8866	0.8645	-	-
5	3	2.0	0.9467	0.9231	0.8740	-	-
6	4	1.5	0.9512	0.9334	0.8878	0.8712	-
7	4	1.5	0.9813	0.9334	0.9159	0.8712	-
8	4	1.5	0.9813	0.9334	0.8878	0.8712	-
9	4	1.5	0.9512	0.9334	0.9159	0.8712	-
10	5	1.2	0.9654	0.9509	0.9180	0.9042	0.8729
11	5	1.2	0.9850	0.9388	0.9247	0.8813	0.8680

**Table 9:** Effective densities and effective percentages by weight of Bi_2_O_3_ for all 11 multi-layered Bi_2_O_3_/NR composites (Sample# denotes Sample Number).

Sample#	Effective Density (g/cm^3^)	Effective Percentage by Weight (wt.%)
1	1.011	10.00
2	1.021	10.98
3	1.021	10.98
4	1.033	12.19
5	1.015	10.47
6	1.021	10.98
7	1.021	10.98
8	1.021	10.98
9	1.021	10.98
10	1.017	10.64
11	1.026	11.55

**Table 10:** Comparative final transmission factors (I/I_0_) of 11 multi-layered structures of Bi_2_O_3_/NR composites at X-ray energies of 50, 100, 150, and 200 keV using XCOM and PHITS and their corresponding percentage differences (Sample# denotes Sample Number).

Sample#	XCOM				PHITS				%Difference			
	Final Transmission Factor (I/I_0_) at X-ray Energy (keV)											
	50	100	150	200	50	100	150	200	50	100	150	200
1	0.5663	0.6671	0.8220	0.8765	0.5732	0.6681	0.8258	0.8785	1.22	0.15	0.46	0.23
2	0.5388	0.6447	0.8119	0.8712	0.5518	0.6483	0.8498	0.8964	2.42	0.55	4.67	2.89
3	0.5388	0.6447	0.8119	0.8712	0.5578	0.6756	0.8196	0.8946	3.54	4.78	0.95	2.69
4	0.5064	0.6178	0.7995	0.8645	0.5289	0.6371	0.8255	0.8865	4.44	3.11	3.25	2.54
5	0.5529	0.6564	0.8172	0.8740	0.5766	0.6589	0.8443	0.8871	4.27	0.38	3.32	1.50
6	0.5388	0.6447	0.8119	0.8712	0.5428	0.6480	0.8226	0.8735	0.75	0.50	1.32	0.27
7	0.5388	0.6447	0.8119	0.8712	0.5494	0.6547	0.8432	0.8898	1.97	1.54	3.85	2.14
8	0.5388	0.6447	0.8119	0.8712	0.5471	0.6452	0.8461	0.8917	1.55	0.08	4.22	2.36
9	0.5388	0.6447	0.8119	0.8712	0.5596	0.6522	0.8480	0.8789	3.87	1.15	4.45	0.89
10	0.5479	0.6522	0.8153	0.8729	0.5550	0.6723	0.8523	0.8861	1.30	3.08	4.54	1.50
11	0.5232	0.6319	0.8060	0.8680	0.5297	0.6384	0.8343	0.9039	1.24	1.03	3.51	4.13

**Table 11:** Values for µ_eff_, µ_m,eff_, and HVL_eff_ of 11 multi-layered Bi_2_O_3_/NR composites at X-ray energies of 50, 100, 150, and 200 keV (Sample# denotes Sample Number).

Sample#	µ_eff_ (cm^−1^)				µ_m,eff_ (cm^2^/g)				HVL_eff_ (cm)			
	50 keV	100 keV	150 keV	200 keV	50 keV	100 keV	150 keV	200 keV	50 keV	100 keV	150 keV	200 keV
1	0.9479	0.6748	0.3267	0.2197	0.9379	0.7195	0.4541	0.4838	0.7313	1.0272	2.1215	3.1549
2	1.0308	0.7315	0.3473	0.2299	1.0101	0.7243	0.4796	0.4793	0.6724	0.9475	1.9956	3.0153
3	1.0308	0.7315	0.3473	0.2299	1.0101	0.7243	0.4796	0.4793	0.6724	0.9475	1.9956	3.0153
4	1.1341	0.8025	0.3730	0.2426	1.0980	0.7309	0.5103	0.4755	0.6112	0.8637	1.8585	2.8569
5	0.9876	0.7017	0.3365	0.2245	0.9727	0.7214	0.4664	0.4814	0.7019	0.9878	2.0599	3.0873
6	1.0308	0.7315	0.3473	0.2299	1.0101	0.7243	0.4796	0.4793	0.6724	0.9475	1.9956	3.0153
7	1.0308	0.7315	0.3473	0.2299	1.0101	0.7243	0.4796	0.4793	0.6724	0.9475	1.9956	3.0153
8	1.0308	0.7315	0.3473	0.2299	1.0101	0.7243	0.4796	0.4793	0.6724	0.9475	1.9956	3.0153
9	1.0308	0.7315	0.3473	0.2299	1.0101	0.7243	0.4796	0.4793	0.6724	0.9475	1.9956	3.0153
10	1.0028	0.7122	0.3403	0.2265	0.9860	0.7224	0.4712	0.4807	0.6912	0.9732	2.0366	3.0608
11	1.0797	0.7650	0.3594	0.2359	1.0520	0.7272	0.4943	0.4773	0.6420	0.9061	1.9284	2.9382
